# No genetic erosion after five generations for *Impatiens glandulifera* populations across the invaded range in Europe

**DOI:** 10.1186/s12863-019-0721-4

**Published:** 2019-02-19

**Authors:** Kenny Helsen, Jenny Hagenblad, Kamal P. Acharya, Jörg Brunet, Sara A. O. Cousins, Guillaume Decocq, Pieter De Frenne, Adam Kimberley, Annette Kolb, Jana Michaelis, Jan Plue, Kris Verheyen, James D. M. Speed, Bente J. Graae

**Affiliations:** 1 Department of Biology, Norwegian University of Science and Technology, Høgskoleringen 5, NO-7034 Trondheim, Norway; 2 Plant Conservation and Population Biology, Biology Department, University of Leuven, Kasteelpark Arenberg 31, BE-3001 Heverlee, Belgium; 3 IFM – Biology, Linköping University, SE-581 83 Linköping, Sweden; 4 Southern Swedish Forest Research Centre, Swedish University of Agricultural Sciences, Box 49, SE-230 53 Alnarp, Sweden; 5 Department of Physical Geography, Stockholm University, SE-106 91 Stockholm, Sweden; 6 Edysan (FRE 3498 CNRS), Centre National de la Recherche Scientifique, Université de Picardie Jules Verne, 1 rue des Louvels, FR-80037 Amiens Cedex, France; 7 Forest & Nature Lab, Ghent University, Geraardsbergsesteenweg 267, BE-9090, Gontrode-Melle, Belgium; 8 Vegetation Ecology and Conservation Biology, Faculty of Biology/Chemistry (FB 02), Institute of Ecology, University of Bremen, Leobener Strasse 5, 28359 Bremen, Germany; 9 Department of Natural History, NTNU University Museum, Norwegian University of Science and Technology, NO-7491 Trondheim, Norway

**Keywords:** Colonization event, Founder effect, Genetic bottleneck, Himalayan balsam, Latitudinal gradient, Population re-establishment, SSRs

## Abstract

**Background:**

The observation that many alien species become invasive despite low genetic diversity has long been considered the ‘genetic paradox’ in invasion biology. This paradox is often resolved through the temporal buildup genetic diversity through multiple introduction events. These temporal dynamics in genetic diversity are especially important for annual invasive plants that lack a persistent seed bank, for which population persistence is strongly dependent on consecutive seed ‘re-establishment’ in each growing season. Theory predicts that the number of seeds during re-establishment, and the levels of among-population gene flow can strongly affect recolonization dynamics, resulting in either an erosion or build-up of population genetic diversity through time. This study focuses on temporal changes in the population genetic structure of the annual invasive plant *Impatiens glandulifera* across Europe. We resampled 13 populations in 6 regions along a 1600 km long latitudinal gradient from northern France to central Norway after 5 years, and assessed population genetic diversity with 9 microsatellite markers.

**Results:**

Our study suggests sufficiently high numbers of genetically diverse founders during population re-establishment, which prevent the erosion of local genetic diversity. We furthermore observe that *I. glandulifera* experiences significant among-population gene flow, gradually resulting in higher genetic diversity and lower overall genetic differentiation through time. Nonetheless, moderate founder effects concerning population genetic composition (allele frequencies) were evident, especially for smaller populations.

Despite the initially low genetic diversity, this species seems to be successful at persisting across its invaded range, and will likely continue to build up higher genetic diversity at the local scale.

**Electronic supplementary material:**

The online version of this article (10.1186/s12863-019-0721-4) contains supplementary material, which is available to authorized users.

## Background

The number of invasive alien species continues to increase across the globe [1, 2]. Consequently, much research has focused on understanding the population genetic processes underlying the successful establishment and spread of invasive alien species outside of their native range [3–5]. This work has clearly shown that during the invasion process, many alien species obtain relatively low levels of genetic diversity [3, 6, 7]. This low genetic diversity is directly caused by the often small number of initial colonists, thus introducing only a small subset of the genetic diversity present in the native range [5, 8]. These genetically poor and small initial populations are further subjected to strong genetic drift or founder effects during the early stages of the invasion process, which may further erode genetic diversity and hamper the invasion success on longer timescales [3, 5, 9].

Range expansion of the invasive species into new areas can result in additional sequential founder effects, bottlenecks and increased genetic drift, further eroding genetic diversity [4, 10]. Several invasive species furthermore seem to show boom-bust dynamics, i.e. the rise of populations to outbreak levels, followed by a dramatic decline, suggesting that these potentially genetically poor populations can crash when certain local selective pressures shift [11, 12]. The observation, however, that many alien species become invasive, despite their expected bottlenecked populations, low genetic diversity and low evolutionary potential, has long been considered the ‘genetic paradox’ in invasion biology [3, 13]. Several chronosequence-based studies have, however, shown that the genetic paradox is often ‘resolved’ through the buildup of higher genetic diversity following multiple introduction events from the native range [6, 10, 13, 14]. This clearly illustrates how temporal dynamics affect genetic diversity patterns of invasive aliens species after initial invasion, which can, in turn, determine the long-term success of these species in their invaded range [3]. Furthermore, since not only the invasive species’ fitness and population persistence, but also its long-term ecosystem impacts and success of potential eradication or control measures are dependent on its population genetic diversity, it is important to understand how these temporal dynamics will affect its population genetic structure [3, 15, 16]. Indeed, if eradication methods can optimize reduction in genetic diversity, the species’ persistence and spread may be minimized due to increased genetic drift effects [5]. Nevertheless, we know very little about the temporal dynamics of population genetic diversity of invasive species, and repeated sampling of the same populations has rarely been done ([16], however see [17–20]).

These temporal dynamics in genetic diversity are particularly important for annual alien invasive (plant) species, where long-term population persistence is strongly dependent on successful seed establishment in each growing season. Depending on the seedling recruitment success (colonists) and the efficiency of long-distance seed dispersal (i.e. an influx of migrants), these re-establishment dynamics could result in sequential genetic founder events and genetic bottlenecks [21, 22]. Indeed, theory predicts that if re-establishment is effectuated by a limited number of individuals (seeds), and only limited among-population gene flow occurs (‘propagule pool’ colonization model sensu [23]), these recolonization dynamics will result in erosion of population genetic diversity and inflation of among-population genetic differentiation through time [9, 17, 24]. Alternatively, if sufficiently high seedling recruitment and high (long-distance) gene flow occur during population re-establishment, these consecutive founder events might retain reasonably high levels of genetic diversity (‘migrant pool’ colonization model sensu [23]). This could even result in a gradual increase in genetic diversity and decrease in across-population genetic differentiation, potentially resulting in stabilization of local population sizes and increase of the overall invasion success of the annual species across its invaded range [17].

Here we focus on the annual invasive alien plant *Impatiens glandulifera* Royle (*Balsaminaceae*) (2n = 18 or 20). This species was originally introduced to Europe in the 1800s as an ornamental plant from the western Himalayas [25] and subsequently colonized riparian habitats across its invaded range from southern Spain (37°N) to northern Norway (70°N) [25, 26]. The species is highly competitive and can affect several ecosystem functions, such as nutrient cycling and soil erosion control [27, 28]. Although *I. glandulifera* can form large populations, the species has strongly fluctuating annual population sizes [17, 29]. These temporal fluctuations in population size and population persistence are mainly caused by the species’ annual lifecycle and the absence of a persistent seed bank [25]. Previous research has observed local adaptation of several life-history traits in this species [30], suggesting sufficiently high genetic diversity (however see [31]). This anticipated high genetic diversity is further supported by the expectation of substantial gene flow within and across populations through both hydrochorous dispersed seeds [32, 33] and pollen [34]. Other studies have nevertheless observed genetically impoverished *I. glandulifera* populations across several parts of its invaded range [7, 35]. Similarly, a recent study showed relatively high genetic differentiation of *I. glandulifera* populations both within and across river catchments in the UK, suggesting founder/drift dynamics due to sequential population re-establishment under limited gene flow [17]. These contradicting genetic results suggest that the temporal genetic dynamics are complex in this species. However, these studies did not evaluate temporal dynamics in population genetic diversity.

In this study, we resampled 13 *I. glandulifera* populations, ten of which were studied in [7], to assess changes in the neutral genetic diversity 5 years after the initial sampling. These populations are distributed across six study regions along a 1600 km long latitudinal gradient in Europe, ranging from Amiens (France) in the south to Trondheim (Norway) in the north. We expect that, if these populations have experienced strong sequential founder effects in the 5 years between sampling years due to increased genetic drift and low gene flow levels, population level genetic diversity will have decreased, and among-population genetic differentiation will have increased. Alternatively, if gene flow retained sufficient levels and sequential population re-establishment was effectuated through genetically diverse founders, we expect population genetic diversity and among-population genetic differentiation to remain constant or, even increase, respectively decrease. We furthermore expect these potential population genetic changes to be dependent on population size, with much stronger potential shifts in genetic diversity and genetic differentiation in small populations.

## Results

### Genetic diversity

**Fig. 1:**
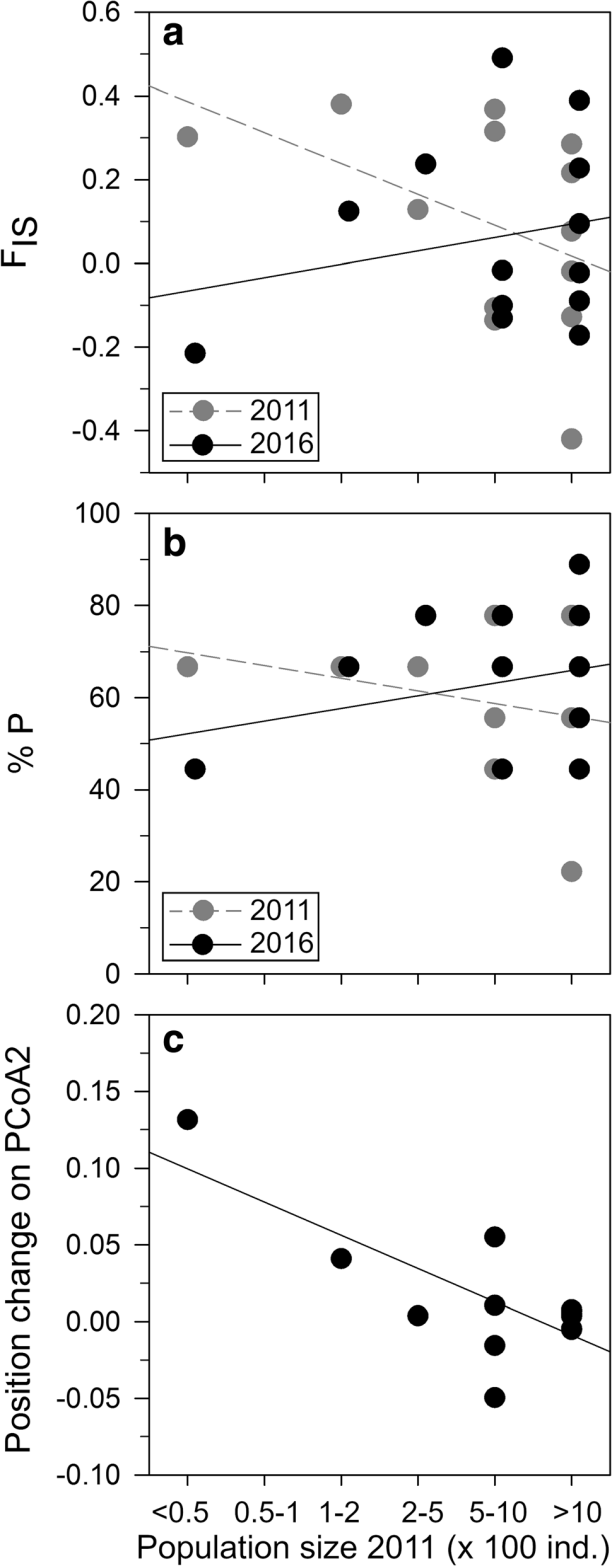
Correlation between genetic diversity measures of *Impatiens glandulifera* populations and the 2011 population size class. **a** the inbreeding coefficient (F_IS_) for the 2011 and 2016 sampling year, **b** percentage of polymorphism (%P) for the 2011 and 2016 sampling year and **c** the change in the second PCoA axis score of each population from the 2011 to 2016 sampling year, based on pairwise F_ST_ values

Population sizes have decreased for almost all populations between the 2011 and 2016 sampling years (Table 1, no decrease in two populations). For 2011, the number of alleles per population (A) varied between 1.1 and 2.2 (average: 1.75), the percentage of polymorphism (%P) varied between 22.2 and 77.8% (average: 59.0%) and the observed heterozygosity (H_O_) varied between 0.09 and 0.26 (average: 0.17) (Table 1). For 2016, A varied between 1.4 and 2.1 (average: 1.76), %P varied between 44.4 and 88.9% (average: 63.2%) and H_O_ varied between 0.07 and 0.25 (average: 0.18) (Table 1). The inbreeding coefficient (F_IS_) was significant for 7 out of 13 populations for the 2011 sampling year, and for 5 out of 13 populations for the 2016 sampling year (Table 1). A, H_O_ and the expected heterozygosity (H_E_) were not significantly different between the two sampling years, nor did they correlate with population size or population size change between 2011 and 2016. F_IS_, however, was significantly lower in 2016 for small populations, but not for large populations according to the repeated measures ANOVA (Fig. 1a) (significant sampling year*2011 population size interaction: F = 7.0, *p* = 0.023; sampling year effect: F = 7.3, *p* = 0.020; 2011 population size effect: F = 0.3, *p* = 0.601). Similarly, the model results for %P showed a slight increase in polymorphism for the large populations but a decrease in the smallest population (Fig. 1b) (sampling year effect: F = 3.3, *p* = 0.096; 2011 population size effect: F < 0.1, *p* = 1.0; sampling year*2011 population size interaction: F = 5.1, *p* = 0.046). Note that these interaction effects for F_IS_ and %P are largely caused by the smallest population ‘Trondheim 3’ (Fig. 1).

**Table 1:** Characteristics of all sampled *Impatiens glandulifera* populations

Study region/ Population	Lat (°N)	Lon (°E)	N_e_	2011						2016					
				pop size	A	H_E_	H_O_	F_IS_	%P	pop size	A	H_E_	H_O_	F_IS_	%P
North France															
Amiens 1^a^	49.922	2.229	8.5	500–1000	1.4	0.19	0.12	0.368^**^	44.4	200–500	1.6	0.13	0.15	−0.131	44.4
Amiens 2^a^	50.014	2.036	5.6	500–1000	1.7	0.15	0.17	−0.106	55.6	100–200	1.7	0.16	0.16	−0.017	44.4
Belgium															
Ghent 1^a^	51.010	3.794	89.0	> 1000	1.9	0.25	0.20	0.216^**^	77.8	> 1000	1.8	0.26	0.25	0.094	77.8
Ghent 2^b^	50.884	3.929	52.7	> 1000	1.2	0.11	0.10	0.076	22.2	200–500	1.8	0.16	0.13	0.227^*^	55.6
Germany															
Bremen 1^a^	53.130	8.786	32.0	> 1000	2.2	0.25	0.26	−0.019	77.8	100–200	1.9	0.24	0.25	−0.023	66.7
Bremen 2^b^	53.164	8.753	162.2	> 1000	1.7	0.22	0.25	−0.128	55.6	500–1000	1.8	0.21	0.25	−0.172	66.7
South Sweden															
Lund 1^a^	55.994	12.800	13.3	100–200	2.1	0.14	0.09	0.380^***^	66.7	50–100	1.8	0.17	0.16	0.124^(*)^	66.7
Lund 2^a^	55.977	12.820	4.7	500–1000	2.1	0.21	0.15	0.315^***^	77.8	200–500	1.9	0.13	0.07	0.490^***^	77.8
Central Sweden															
Stockholm 1^a^	59.163	18.168	35.7	200–500	2.0	0.26	0.24	0.128^(*)^	66.7	100–200	2.1	0.24	0.19	0.237^**^	77.8
Stockholm 2^b^	59.409	17.860	41.9	500–1000	1.8	0.16	0.18	−0.136	55.6	200–500	1.7	0.17	0.20	−0.101	66.7
Central Norway															
Trondheim 1^a^	63.479	10.999	56.9	> 1000	1.8	0.23	0.17	0.285^**^	77.8	200–500	2.0	0.28	0.19	0.389^***^	88.9
Trondheim 2^a^	63.477	10.964	10.5	> 1000	1.1	0.09	0.12	−0.420	22.2	200–500	1.4	0.14	0.16	−0.090	44.4
Trondheim 3^a^	63.413	10.809	4.2	< 50	1.8	0.18	0.14	0.302^**^	66.7	50–100	1.6	0.17	0.21	−0.215	44.4

Information about location, effective population size (Ne), actual population size (number of individuals), mean number of alleles (A), expected heterozygosity (H_E_), observed heterozygosity (H_O_), inbreeding coefficient (F_IS_) and percentage of polymorphism (%P) for each population in both the 2011 and 2016 sampling year. Significance: ^(*)^: 0.10 ≥ *P*-value > 0.05; ^*^: 0.05 ≥ *P*-value > 0.01; ^**^: 0.01 ≥ *P*-value > 0.001; ^***^: 0.001 ≥ *P*-value

^a^2011 population information originates from the Hagenblad et al. [7] study

^b^2011 population information obtained from new genetic analyses on stored leaf material

### Genetic bottlenecks and effective population size

We found evidence for a small heterozygosity excess, indicating recent bottlenecks in population Bremen 2 (Wilcoxon *p* = 0.047) and Amiens 1 (Wilcoxon *p* = 0.031) for 2011. For the 2016 data, however, no significant recent bottlenecks were detected. As expected considering the low overall genetic diversity of the species, the assessed effective population sizes were relatively small, and much smaller than the actual population sizes (average: 39.8, range: 4.2–162.2) (Table 1).

### Genetic differentiation

**Fig. 2:**
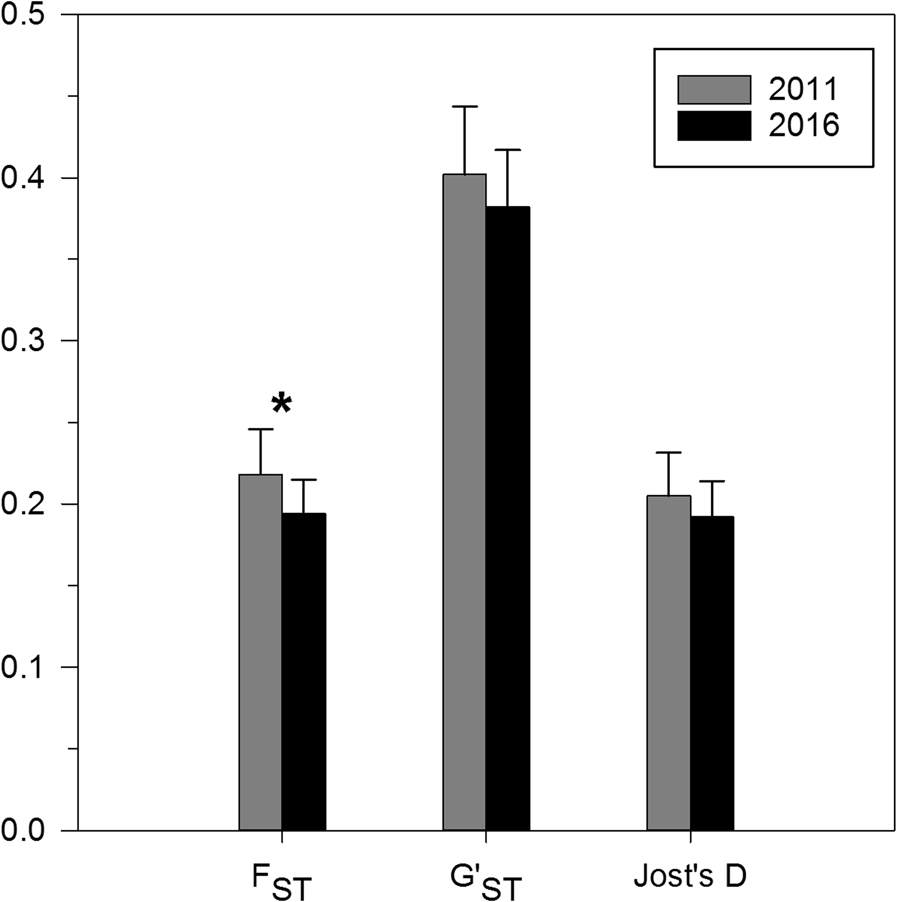
Differences in mean pairwise *Impatiens glandulifera* population genetic differentiation between 2011 and 2016. Genetic differentiation based on F_ST_, G’_ST_ and Jost’s D. 95% bootstrap confidence intervals given. *: significant difference between the 2011 and 2016 sampling year

Genetic differentiation was significant among all pairwise populations for each sampling year, except between population Ghent 1 and population Ghent 2 (see Additional files 1, 2, 3 and 4). Genetic differentiation was furthermore significant between both sampling years for 9 out of 13 populations based on F_ST_ (average: 0.048, range: 0.007–0.137) and for 10 out of 13 populations based on G’_ST_ (average: 0.076, range: − 0.007–0.271) and Jost’s D (average: 0.048, range: − 0.006–0.116) (see Additional files 1, 2 and 3). Pairwise genetic differentiation among populations was significantly lower in 2016 than in 2011 based on F_ST_, but was not significantly different based on G’_ST_ and Jost’s D (Table 2, Fig. 2). The analysis for null-allele corrected F_ST_ also indicated significantly lower genetic differentiation in 2016 (results not shown).

**Table 2:** Parameter estimates of bootstrapping paired t-tests on genetic differentiation between 2011 and 2016 populations

	2011 pop.		2016 pop.		t-test	
	Mean	CI	Mean	CI	Mean difference	CI
F_ST_	0.218	0.191–0.247	0.194	0.173–0.215	0.025^**^	0.007–0.042
G’_ST_	0.402	0.361–0.444	0.382	0.346–0.416	0.021	−0.010–0.050
Jost’s D	0.205	0.179–0.232	0.192	0.170–0.214	0.013	−0.003–0.028

Genetic differentiation means, mean differences and confidence intervals for paired t-tests on pairwise genetic differentiation for 2011 and 2016 *Impatiens glandulifera* populations. All tests are based on 9999 bootstraps. CI: 95% bootstrap confidence intervals. Significance: ^**^: 0.01 ≥ *P*-value > 0.001

The analysis of molecular variance (AMOVA) indicated that molecular variance was significantly partitioned across all tested hierarchic levels for both sampling years (*p* < 0.001) (Table 3). Although the percentage of molecular variance among study regions remained constant (26%), the percentages declined among populations (21 to 19%) and among individuals (16 to 12%), with a proportional increase in the percentage of molecular variance within individuals (37 to 43%) (Table 3).

**Table 3:** Results of AMOVA’s on genetic differentiation for the *Impatiens glandulifera* populations in 2011 and 2016

	2011 pop.			2016 pop.		
	F-stat	mol. var.	% mol. var.	F-stat	mol. var.	% mol. var.
Among study regions (F_RT_)	0.263^***^	0.527	26	0.263^***^	0.494	26
Among populations (F_SR_)	0.279^***^	0.413	21	0.264^***^	0.366	19
Among individuals (F_ST_)	0.469^***^	0.315	16	0.458^***^	0.218	12
Within individuals (F_IS_)	0.296^***^	0.751	37	0.213^***^	0.803	43
Total (F_IT_)	0.626^***^	2.006	100	0.573^***^	1.881	100

Analysis includes all populations along the latitudinal gradient from Amiens, France to Trondheim, Norway for the 2011 and 2016 sampling separately. F-statistics and molecular variance provided for each nested level. All tests are based on 9999 permutations. Significance: ^***^: 0.001 ≥ *P*-value

**Fig. 3:**
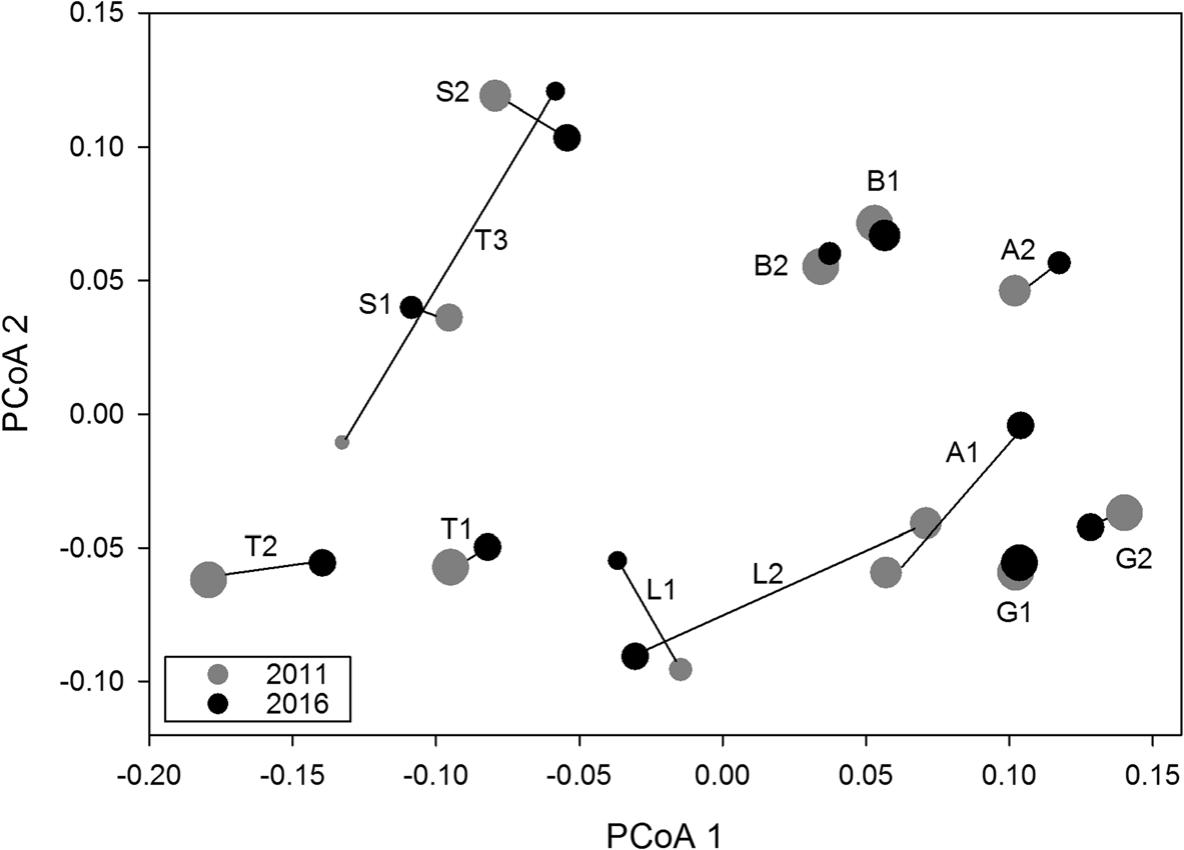
Principle coordinates analysis (PCoA) based on the pairwise F_ST_ matrix. Lines connect each population of *Impatiens glandulifera* from the 2011 (grey circle) and 2016 (black circle) sampling year. Population codes at connection lines: A = Amiens, G = Ghent, B=Bremen, L = Lund, S=Stockholm, T = Trondheim. Population circle sizes corresponds to population size groups (ordinal levels) given in Table 1. The first three PCoA axes explained 35.29, 17.16 and 13.81% of the total variation, respectively

Results of the principle coordinate analysis showed relatively large changes in genetic makeup for several populations between the 2011 and 2016 sampling year (Fig. 3). The repeated measures ANOVAs showed that part of this change was related to initial population size. More specifically changes on PCoA axis 2 between both sampling years were strongest for small populations (Fig. 1c) (significant sampling year*2011 population size interaction: F = 14.5, *p* = 0.003; sampling year effect: F = 17.3, *p* = 0.002; 2011 population size effect: F = 0.3, *p* = 0.591). Note that this pattern was mainly caused by the smallest population ‘Trondheim 3’ (Fig. 1c). Changes in PCoA axes 1 and 3 were not mediated by initial population size (results not shown). PCoA and subsequent repeated measures ANOVA based on null-allele corrected F_ST_ showed similar results as those based on uncorrected F_ST_ (results not shown).

## Discussion

The overall constant genetic diversity in both sampling years, slightly increased polymorphism and reduced among-population genetic differentiation, strongly suggest that population re-establishment is effectuated by relatively high numbers of colonists and migrants, thus stabilizing both genetic diversity and among-population genetic differentiation across generations. These results are in agreement with observed temporal genetic patterns in two short-lived invasive alien insects after tens of generations [18, 19]. In other words, despite the initially low genetic diversity and associated low effective population sizes, *I. glandulifera* seems to be successful at persisting across its invaded range seemingly due to surprisingly high levels of gene flow. Our results thus support the theoretical ‘migrant pool’ colonization model, rather than the ‘propagule pool’ colonization model of annual population re-establishment between 2011 and 2015 [23, 24].

Although no evidence was found for genetic bottlenecks in 2016 and slight increases in genetic diversity were evident, substantial shifts in the genetic makeup did occur for most populations. This is in agreement with the observed temporal genetic differentiation for two *I. glandulifera* populations after two generations in the UK [17]. These results suggest that, although relatively stable in genetic diversity, these populations do experience significant founder effects and potential drift in their genetic makeup (allele frequencies) during sequential population re-establishment [17].

As previously observed, overall population genetic diversity was low across the invaded European range of *I. glandulifera* in 2011 [7], which is in line with the often observed low genetic diversity and heterozygosity for invasive alien species in their invaded range [3, 6, 7]. We furthermore detected significant inbreeding coefficients for seven of the 13 studied populations in 2011, contradictory to the results of Hagenblad et al. [7]. Note that these differences in F_IS_ between both studies are likely caused by the different number of studied individuals per population (23 in this study vs. 30 in [7]) and the different number of studied populations (13 vs. 10). However, high inbreeding coefficients (> 0.20) have previously been observed for this species in different regions of both its invaded and native range [17, 35]. In our study, high F_IS_ values are likely caused by selfing and biparental inbreeding, as a direct consequence of the overall low genetic diversity. However, a Wahlund effect might also be partly responsible for the high F_IS_ values, if subpopulation structure arises due to population re-establishment of a mixture of natural founders and recent colonists [17, 36].

Interestingly, the retention of genetic diversity during sequential population re-establishment is not solely driven by high local seed colonization. Indeed, the significant decrease in overall genetic differentiation, decreased among-population molecular variance (F_SR_) and increased polymorphism/within-individual molecular variance (F_IS_) all suggests that gene flow among (local) populations is significantly shaping genetic patterns of these populations. This illustrates how *I. glandulifera*’s potential for within-study region, long-distance (mainly hydrochorous) seed dispersal can contribute to this species’ temporal genetic patterns.

Not surprisingly considering the geographical distances, no indication for among-region gene flow was observed. The among-region molecular variance (F_RT_) has remained constant at the relatively high 26% level between both sampling years, also observed in the relatively high, average (among-region) genetic differentiation levels (F_ST_ = 0.218). This furthermore helps to explain why local (within-region) population genetic diversity has remained so low, despite the presence of among-population gene flow and strong differences in allele frequencies and identities among study regions [7]. We can, however, expect that this pattern is temporary. Indeed, over longer time scales, long-distance gene flow will very likely result in the mixing of genetic material of the different areas along the invaded range, thus gradually increasing overall genetic diversity and potentially fitness of local populations [13]. This scenario is equivalent to the sequential introduction events with subsequent gene pool mixing that has been observed for several invasive alien species [6, 10, 14]. Also note that the occurrence of populations with high genetic diversity in different parts of *I. glandulifera*’s invaded range, such as Finland [35] and Lithuania [37], might be partly caused by such gene flow and subsequent gene pool mixing events. Especially the mixing of the genetically very dissimilar Stockholm populations [7], with the more southern populations could result in strong increases in local population genetic diversity.

Despite the retention of genetic diversity at the population level, all but one of the resampled populations decreased in size between 2011 and 2016. Although part of this decline might be due to local eradication actions, this reduction could also reflect temporary population size fluctuations, incidentally due to suboptimal weather conditions in spring of 2016. To really assess if there is a consistent temporal trend toward population size reduction across the gradient, population sizes should be assessed consecutively in the following years.

Initial population sizes affected several patterns, although effects were mainly caused by one outlier (the single small population ‘Trondheim 2’ in our dataset). Our results showed that initially small populations were characterized by the largest shifts, both in genetic composition and in F_IS_, possibly caused by the combined actions of increased genetic drift and higher chances of founder effects in small populations. This likely illustrates the importance of large population sizes and associated high seed production to overcome deleterious effects of sequential population re-establishment in this species [23, 38]. These effects of population size only seem to become important below a certain population size threshold however, since the reduction in population size between 2011 and 2016 for most populations did not affect any of the genetic patterns. Alternatively, population size effects might be partly masked by potential substantial annual population size fluctuations during the last 5 years. Indeed, this species is known to occasionally exhibit large fluctuations in annual population sizes [17, 29]. Additionally, although no persistent seed bank exists, research has shown that, at least some seeds can persist up to 2 years in the soil [25]. Consequently, germination of these older seeds during population re-establishment, can likely moderately buffer the deleterious genetic effects of large population size fluctuations. Evaluation of temporal genetic patterns for additional, initially small populations could assess the validity of our current results regarding the importance of population size. Effective population sizes were nonetheless extremely small for all populations, suggesting that population re-establishment is likely occurring through many seeds originating from only a limited number of (genetically) different plant individuals, which is not surprising considering the high fecundity of most *I. glandulifera* plants [25].

## Conclusions

In sum, we observed a small temporal increase in genetic diversity and decrease in among-population genetic differentiation between 2011 and 2016, for several *I. glandulifera* populations across Europe, despite a seemingly overall decrease in their population sizes. These results suggest that annual population re-establishment is following the ‘migrant pool’ colonization model [23], thus preventing the erosion of local genetic diversity and inflation of among-population genetic differentiation through the combined action of genetic bottlenecks and drift [9, 24]. Our results do nonetheless suggest moderate founder effects concerning population genetic composition (allele frequencies), especially for smaller populations, which is in agreement with the results of Walker et al. [17].

Our study furthermore suggests that *I. glandulifera* experiences significant among-population gene flow, gradually resulting in higher genetic diversity and lower overall genetic differentiation. Despite the initially low genetic diversity and associated low effective population sizes, this species seems to be successful at persisting across its invaded range, and will likely continue to build up higher genetic diversity at the local scale, potentially further enhancing its success. These results suggest that it is very unlikely that this species will show boom-bust dynamics on the longer run, despite its tendency for strong population size fluctuations [29]. In other words, if the species is to be removed from its invaded range, this will have to be effectuated through active eradication measures, since genetically-driven local extinctions are unlikely. The results furthermore suggest that long-term fitness and adaptive potential of this species will likely continue to rise across the invasive range, due to slow but gradual increase in local genetic diversity. This could result in more pronounced population persistence and potential expansion of its current invaded range across Europe.

## Methods

### Sampling and laboratory procedures

In 2011, six *Impatiens glandulifera* populations with at least 30 flowering individuals were selected for each of six study regions along a 1600 km latitudinal European gradient, ranging from Amiens (France) in the south to Trondheim (Norway) in the north (for more information see: [7, 30]) (Table 1). Each population was defined as a single continuous patch of *I. glandulifera* individuals. Populations were mainly located in wet areas, in forests or on forest edges, often in the vicinity of waterways. Within each municipality, all six populations were sampled with a minimum distance of 1.8 km between each population. Leaf material was collected from 30 random individuals for each population in 2011 and stored after 24 h drying at 45 °C. All populations were revisited in 2016 with the help of GPS coordinates and new leaf samples were collected for 30 random individuals and dried using silica gel. Conform the 2011 sampling campaign, 2016 sampling was performed according to national legislations [7]. Population size was assessed during both sampling years using six ordinal levels; 1. < 50 individuals; 2. 50–100 ind.; 3. 100–200 ind.; 4. 200–500 ind.; 5. 500–1000 ind.; 6. > 1000 ind. More specifically, we counted up to 100–200 individual plants, and consequently used the patch size of this counted part to visually assess the approximate number of individuals of the whole population. The change in population size between both sampling years was calculated as the difference between the 2011 and 2016 population size ordinal level. Note that sampling and population size estimations for 2016 were performed in collaboration with the original collectors, using the detailed written protocols from the 2011 sampling campaign.

We used the *I. glandulifera* individuals of the ten European populations that had been microsatellite genotyped in the study of Hagenblad et al. [7], named Amiens 1 & 2, Ghent 1, Bremen 1, Lund 1 & 2, Stockholm 1 and Trondheim 1, 2 & 3 (Table 1). We genotyped an additional three 2011 populations (Ghent 2, Bremen 2 and Stockholm 2, Table 1), using stored dried leaf samples, resulting in a total of two genotyped populations for each study region, except the Trondheim region, where three populations were genotyped (Table 1). The same 13 populations were genotyped for the 2016 samples. Due to logistic constraints only 23 randomly selected individuals of the collected 30 were genotyped for each population. Consequently, 23 individuals were also randomly selected for each population from the original Hagenblad et al. [7] dataset. This setup resulted in a total of 598 genotyped individuals across 13 populations and two time-points (2011 and 2016).

We used E.Z.N.A HP plant DNA mini kits for leaf DNA extraction (Omega Bio-tek Inc., GA, USA). We amplified nine microsatellites previously used for the *I. glandulifera* samples from 2011 [7]. Six of these microsatellites were developed by Provan et al. [39] (IGNSSR 101, 104, 203, 210, 213 & 240) and three were developed by Walker et al. [17] (A 2, 21 & 3). We constructed three multiplexes of three to five microsatellites in 10 μl reactions for amplifications. Each multiplex contained 1 μl template DNA, 1.2–2.0 μl of one of the multiplexed primer combinations (50–100 nM primer concentrations), 1.8–2.0 μl RNAse-free water and 5 μl Qiagen Multiplex PCR Master Mix. The PCR cycling profile consisted of an initial denaturation (15 min) at 94 °C, 30 cycles of 30 s at 94 °C, 90 s at 55 °C and 60 s at 72 °C, and final extension (10 min) at 72 °C [7]. After PCR, fragments were sized on a 3130xl Genetic Analyzer (Applied Biosystems, CA, USA) on a mixture of 1 μl PCR reaction, 0.15 μl Applied Biosystems’ GeneScan 500 LIZ size standard and 9.35 μl formamide. The sized fragments were subsequently scored with GeneMapper Software v4.0 (Applied Biosystems, CA, USA).

Initial comparison of the allele identities and frequencies of the newly genotyped individuals, with those of the genotypes obtained by Hagenblad et al. [7], suggested a consistent allele shift between the datasets, potentially due to the use of a different size standard (GeneScan 500 LIZ vs. GeneScan 600 LIZ respectively) [40]. Ten individuals of the Hagenblad et al. [7] dataset, selected to contain 85% of the observed alleles, were subsequently reanalyzed with the described PCR protocol using the original DNA extracts. This data indeed showed a consistent allele shift of two base pairs across all tested alleles, and was subsequently used to calibrate all genotype data to the original Hagenblad et al. [7] standardized allele identities [40]. Twenty individuals of the 2016 sampling year were furthermore genotyped twice, with an overall reproducibility of 98% of the genotypic allele patterns.

### Data analysis

#### Genetic diversity

We used Micro-Checker to assess potential problems with scoring errors due to null alleles, stutter bands or large allele dropout [41]. Although no stutter bands and large allele dropouts were observed, Micro-Checker did indicate a homozygote excess (potential null alleles) for six different loci in at least one, and up to eight, of the 13 populations in at least one of the two time-points. However, considering the overall low genetic diversity, we believe that these patterns are likely not caused by null alleles, except for marker IGNSSR101 & A2, which failed to amplify for all individuals of, at least, one population (populations Trondheim 1 and Trondheim 2, respectively). This is further supported by the observation of high estimated null allele frequencies (> 30%) for IGNSSR101 & A2, but not the other markers, following the Expectation Maximization algorithm for null allele frequency estimation [42] with the FreeNA software [43]. Both markers were nonetheless included for the genetic diversity measures, since exclusion resulted in comparable results for genetic diversity (results not shown).

We calculated the mean number of alleles (further referred to as “A”), observed heterozygosity (H_O_), expected heterozygosity (H_E_) and polymorphism (%P, the percentage of polymorphic loci across all loci) for each population using GenAlEx 6.503 [44]. The inbreeding coefficient (F_IS_) was estimated for all populations based on Weir & Cockerham’s *F*-statistics [45] and significance levels were inferred using 9999 permutations of alleles among individuals within populations with FSTAT 2.9.3 [46].

To test for changes in genetic diversity between the two sampling years we used repeated measures ANOVA (Pillai’s Trace test) with sampling year as repeated measure factor and 2011 population size, change in population size between 2011 and 2016 and their interaction as covariates using A, H_O_, H_E_, F_IS_ and %P as dependent variables *(SPSS Statistics 21.0)*. Final models were obtained using backward model selection on the covariates based on *p*-values.

#### Genetic bottlenecks and effective population size

We used the Bottleneck software to test for potential recent bottleneck events in each population at both sampling years, using the two-phase model of mutation (TPM) with a 90% stepwise component [47]. This technique tests for bottleneck events by looking for evidence of excess heterozygosity relative to allele numbers [47]. Effective population size (N_e_) was assessed using the temporal method, for which the genetic composition of each population across the two sampling years (five generations) is used to estimate N_e_. More specifically we used the method of Jorde and Ryman [48], which is considered more appropriate for small sample sizes and skewed allele frequencies compared to more classical methods which often overestimate N_e_. N_e_ estimations were furthermore based on plan I sampling (non-destructive sampling), with a 0.02 critical value (frequency) for rare allele exclusion, using the NeEstimator v2 software [49].

#### Genetic differentiation

We calculated pairwise genetic differentiation among populations for both sampling years separately, based on Wright’s *F*-statistics (*F*_ST_). Additionally, genetic differentiation was assessed between the two sampling years for each population. We additionally calculated Hedrick’s G’_ST_ and Jost’s D as measures of genetic differentiation, since, unlike F_ST_, these measures are not affected by marker variability [50]. G’_ST_ is the original G_ST_ as defined by Nei [51] standardized by its maximum value [52]. Jost’s D is based on the effective number of alleles rather than on heterozygosity [53]. We used GenAlEx 6.503 for calculation and significance testing (9999 permutations) of all pairwise genetic differentiation metrics [44]. Since two microsatellite markers (IGNSSR101 & A2) failed to amplify for one population, both were excluded for the calculation of all genetic differentiation measures. Additionally, we calculated pairwise F_ST_ values corrected for null-alleles using the ENA (excluding null alleles) correction method with the FreeNA software [43].

We used paired t-tests to compare pairwise among population genetic differentiation (F_ST_, G’_ST_, Jost’s D and null-allele corrected F_ST_) between the 2011 and 2016 populations. Significance of these paired t-tests was assessed based on *9999 bootstraps, to overcome issues with* the pairwise dependency of the data *(SPSS Statistics 21.0)*. We performed a hierarchical analysis of molecular variance (AMOVA) on pairwise F_ST_ values (9999 permutations) with GenAlEx 6.503 [44], for each sampling year (2011 and 2016) separately. AMOVA portioned the total genetic diversity among the six study regions (among-regions), among populations within regions and among individuals within populations.

Genetic differentiation between populations was furthermore visualized using a covariance-based principal coordinates analysis (PCoA) based on the standardized F_ST_-matrix. To test for systematic changes in population-level genetic composition between the two sampling years we used the previously described repeated measures ANOVA design with the plot location on each of the first three PCoA axes as dependent variables *(SPSS Statistics 21.0)*. In these models, sampling year was included as a repeated measure factor and 2011 population size and change in population size between 2011 and 2016 as covariates. A similar PCoA and subsequent repeated measures ANOVA was subsequently performed on the null-allele corrected F_ST_ values.

## Additional files

Additional file 1: Pairwise genetic differentiation among *Impatiens glandulifera* populations (F_ST_). Lower left triangle, F_ST_ estimates for 2011; Upper right triangle, F_ST_ estimates for 2016; values on the main diagonal (grey), F_ST_ estimates between 2011 and 2016 populations along a gradient from Amiens to Trondheim. A = Amiens, G = Ghent, B=Bremen, L = Lund, S=Stockholm, T = Trondheim. Significance: ^NS^: not significant; ^*^: 0.05 ≥ *P*-value > 0.01; ^**^: 0.01 ≥ *P*-value > 0.001; ^***^: 0.001 ≥ *P*-value. (DOCX 15 kb)

Additional file 2: Pairwise genetic differentiation among *Impatiens glandulifera* populations (G’_ST_). Lower left triangle, G’_ST_ estimates for 2011; Upper right triangle, G’_ST_ estimates for 2016; values on the main diagonal (grey), G’_ST_ estimates between 2011 and 2016 populations along a gradient from Amiens to Trondheim. A = Amiens, G = Ghent, B=Bremen, L = Lund, S=Stockholm, T = Trondheim. Significance: ^NS^: not significant; ^*^: 0.05 ≥ *P*-value > 0.01; ^**^: 0.01 ≥ *P*-value > 0.001; ^***^: 0.001 ≥ *P*-value. (DOCX 15 kb)

Additional file 3: Pairwise genetic differentiation among *Impatiens glandulifera* populations (Jost’s D). Lower left triangle, Jost’s D estimates for 2011; Upper right triangle, Jost’s D estimates for 2016; values on the main diagonal (grey), Jost’s D estimates between 2011 and 2016 populations along a gradient from Amiens to Trondheim. A = Amiens, G = Ghent, B=Bremen, L = Lund, S=Stockholm, T = Trondheim. Significance: ^NS^: not significant; ^*^: 0.05 ≥ *P*-value > 0.01; ^**^: 0.01 ≥ *P*-value > 0.001; ^***^: 0.001 ≥ *P*-value. (DOCX 15 kb)

Additional file 4: Pairwise genetic differentiation among *Impatiens glandulifera* populations (Null-allele corrected F_ST_). Lower left triangle, null-allele corrected F_ST_ estimates for 2011; Upper right triangle, null-allele corrected F_ST_ estimates for 2016; values on the main diagonal (grey), null-allele corrected F_ST_ estimates between 2011 and 2016 populations along a gradient from Amiens to Trondheim. A = Amiens, G = Ghent, B=Bremen, L = Lund, S=Stockholm, T = Trondheim. (DOCX 14 kb)

## Abbreviations

A: Number of alleles per population
AMOVA: Analysis of molecular variance
ANOVA: Analysis of variance
CI: Confidence interval
F_IS_: Inbreeding coefficient
F_RT_: Among region molecular variance
F_SR_: Among population molecular variance
H_E_: Expected heterozygosity
H_O_: Observed heterozygosity
N_e_: Effective population size
%P: Percentage of polymorphism
PCoA: Principle coordinate analysis
TPM: Two-phase model of mutation

## References

[1] van Kleunen M, Dawson W, Essl F, Pergl J, Winter M, Weber E (2015). Global exchange and accumulation of non-native plants. Nature.

[2] Chapman D, Purse BV, Roy HE, Bullock JM (2017). Global trade networks determine the distribution of invasive non-native species. Glob Ecol Biogeogr.

[3] Dlugosch KM, Parker IM (2008). Founding events in species invasions: genetic variation, adaptive evolution, and the role of multiple introductions. Mol Ecol.

[4] Lawson Handley L-J, Estoup A, Evans DM, Thomas CE, Lombaert E, Facon B (2011). Ecological genetics of invasive alien species. BioControl.

[5] Sakai AK, Allendorf FW, Holt JS, Lodge DM, Molofsky J, With KA (2001). The population biology of invasive species. Annu Rev Ecol Syst.

[6] Ficetola GF, Bonin A, Miaud C (2008). Population genetics reveals origin and number of founders in a biological invasion. Mol Ecol.

[7] Hagenblad J, Hülskötter J, Acharya KP, Brunet J, Chabrerie O, Cousins SAO (2015). Low genetic diversity despite multiple introductions of the invasive plant species *Impatiens glandulifera* in Europe. BMC Genet.

[8] Nei M, Maruyama T, Chakraborty R (1975). The bottleneck effect and genetic variability in populations. Evolution.

[9] Ingvarsson PK (1997). The effect of delayed population growth on the genetic differentiation of local populations subject to frequent extinctions and recolonisations. Evolution.

[10] Genton BJ, Shykoff JA, Giraud T (2005). High genetic diversity in French invasive populations of common ragweed, *Ambrosia artemisiifolia*, as a result of multiple sources of introduction. Mol Ecol.

[11] Strayer DL, D’Antonio CM, Essl F, Fowler MS, Geist J, Hilt S (2017). Boom-bust dynamics in biological invasions: towards an improved application of the concept. Ecol Lett.

[12] Simberloff D, Gibbons L (2004). Now you see them, now you don’t! - population crashes of established introduced species. Biol Invasions.

[13] Frankham R (2005). Resolving the genetic paradox in invasive species. Heredity.

[14] Valliant MT, Mack RN, Novak SJ (2008). Introduction history and population genetics of the invasive grass *Bromus tectorum* (Poaceae) in Canada. Am J Bot.

[15] Cuddington K, Hastings A (2016). Autocorrelated environmental variation and the establishment of invasive species. Oikos.

[16] Ward S (2006). Genetic analysis of invasive plant populations at different spatial scales. Biol Invasions.

[17] Walker NF, Hulme PE, Hoelzel AR (2009). Population genetics of an invasive riparian species, *Impatiens glandulifera*. Plant Ecol.

[18] Chen YH, Berlocher SH, Opp SB, Roderick GK (2010). Post-colonization temporal genetic variation of an introduced fly, *Rhagoletis completa*. Genetica.

[19] Yang X-M, Lou H, Sun J-T, Zhu Y-M, Xue X-F, Hong X-Y (2015). Temporal genetic dynamics of an invasive species, *Frankliniella occidentalis* (Pergande), in an early phase of establishment. Sci Rep.

[20] Zalewski A, Zalewska H, Lunneryd SG, André C, Mikusiñski G (2016). Reduced genetic diversity and increased structure in American mink on the Swedish coast following invasive species control. PLoS One.

[21] McCauley DE (1991). Genetic consequences of local population extinction and recolonization. Trends Ecol Evol.

[22] Wade MJ, McCauley DE (1988). Extinction and recolonization: their effects on the genetic differentiation of local populations. Evolution.

[23] Slatkin M (1977). Gene flow and genetic drift in a species subject to frequent local extinctions. Theor Popul Biol.

[24] Pannell JR, Charlesworth B (2000). Effects of metapopulation processes on measures of genetic diversity. Philos Trans R Soc Lond Ser B Biol Sci.

[25] Beerling DJ, Perrins JM (1993). Biological flora of the British Isles. *Impatiens glandulifera* Royle (*Impatiens roylei* Walp.). J Ecol.

[26] Beerling DJ (1993). The impact of temperature on the northern distribution limits of the introduced species *Fallopia japonica* and *Impatiens glandulifera* in North-West Europe. J Biogeogr.

[27] Dassonville N, Vanderhoeven S, Vanparys V, Hayez M, Gruber W, Meerts P (2008). Impacts of alien invasive plants on soil nutrients are correlated with initial site conditions in NW Europe. Oecologia.

[28] Greenwood P, Kuhn NJ (2014). Does the invasive plant, *Impatiens glandulifera*, promote soil erosion along the riparian zone? An investigation on a small watercourse in Northwest Switzerland. J Soils Sediments.

[29] Kasperek G (2004). Fluctuations in numbers of neophytes, especially *Impatiens glandulifera*, in permanent plots in a west German floodplain during 13 years. Neobiota.

[30] Acharya KP (2014). Invasive species: genetics, characteristics and trait variation along a latitudinal gradient. PhD dissertation.

[31] Dlugosch KM, Parker IM (2008). Invading populations of an ornamental shrub show rapid life history evolution despite genetic bottlenecks. Ecol Lett.

[32] Love HM, Maggs CA, Murray TE, Provan J (2013). Genetic evidence for predominantly hydrochoric gene flow in the invasive riparian plant *Impatiens glandulifera* (Himalayan balsam). Ann Bot.

[33] Chapman DS, Gray A (2012). Complex interactions between the wind and ballistic seed dispersal in *Impatiens glandulifera* (Royle). J Ecol.

[34] Chittka L, Schürkens S (2001). Successful invasion of a floral market: an exotic Asian plant has moved in on Europe’s river-banks by bribing pollinators. Nature.

[35] Nagy A-M, Korpelainen H (2015). Population genetics of Himalayan balsam (*Impatiens glandulifera*): comparison of native and introduced populations. Plant Ecol Divers.

[36] Helsen K, Jacquemyn H, Honnay O (2015). Hidden founder effects: small-scale spatial genetic structure in recently established populations of the grassland specialist plant *Anthyllis vulneraria*. Mol Ecol.

[37] Zybartaite L, Zukauskiene J, Jodinskiene M, Janssens SB, Paulauskas A, Kupcinskiene E (2011). RAPD analysis of genetic diversity among Lithuanian populations of *Impatiens glandulifera*. Žemdirbystė= Agric.

[38] McCauley DE (1997). The relative contributions of seed and pollen movement to the local genetic structure of *Silene alba*. J Hered..

[39] Provan J, Love HM, Maggs CA (2007). Development of microsatellites for the invasive riparian plant *Impatiens glandulifera* (Himalayan balsam) using intersimple sequence repeat cloning: primer note. Mol Ecol Notes.

[40] Ellis JS, Gilbey J, Armstrong A, Balstad T, Cauwelier E, Cherbonnel C (2011). Microsatellite standardization and evaluation of genotyping error in a large multi-partner research programme for conservation of Atlantic salmon (*Salmo salar* L.). Genetica.

[41] Van Oosterhout C, Hutchinson WF, Wills DPM, Shipley P (2004). Micro-checker: software for identifying and correcting genotyping errors in microsatellite data. Mol Ecol Notes.

[42] Dempster AP, Laird NM, Rubin DB (1977). Maximum likelihood from incomplete data via the EM algorithm. J R Stat Soc B.

[43] Chapuis M-P, Estoup A (2007). Microsatellite null alleles and estimation of population differentiation. Mol Biol Evol.

[44] Peakall R, Smouse PE (2006). GENALEX 6: genetic analysis in excel. Population genetic software for teaching and research. Mol Ecol Notes.

[45] Weir BS, Cockerham CC (1984). Estimating F-statistics for the analysis of population structure. Evolution.

[46] Goudet J (1995). FSTAT (version 1.2): a computer program to calculate F-statistics. J Hered.

[47] Cornuet JM, Luikart G (1996). Description and power analysis of two tests for detecting recent population bottlenecks from allele frequency data. Genetics.

[48] Jorde PE, Ryman N (2007). Unbiased estimator for genetic drift and effective population size. Genetics.

[49] Do C, Waples RS, Peel D, Macbeth GM, Tillett BJ, Ovenden JR (2014). NeEstimator v2: re-implementation of software for the estimation of contemporary effective population size (ne) from genetic data. Mol Ecol Resour.

[50] Meirmans PG, Hedrick PW (2011). Assessing population structure: F_ST_ and related measures. Mol Ecol Resour.

[51] Nei M (1973). Analysis of gene diversity in subdivided populations. Proc Natl Acad Sci U S A.

[52] Hedrick PW (2005). A standardized genetic differentiation measure. Evolution.

[53] Jost L (2008). G_ST_ and its relatives do not measure differentiation. Mol Ecol.

