# Supplementary material for: No genetic erosion after five generations for Impatiens glandulifera populations across the invaded range in Europe
Source: BMC Genet. 2019 Feb 19;20:20. doi: 10.1186/s12863-019-0721-4 (PMC6379953; doi:10.1186/s12863-019-0721-4)
Supplement: Supplementary file 1 — Pairwise genetic differentiation among Impatiens glandulifera populations (FST). Lower left triangle, FST estimates for 2011; Upper right triangle, FST estimates for 2016; values on the main diagonal (grey), FST estimates between 2011 and 2016 populations along a gradient from Amiens to Trondheim. A = Amiens, G = Ghent, B=Bremen, L = Lund, S=Stockholm, T = Trondheim. Significance: NS: not significant; *: 0.05 ≥ P-value > 0.01; **: 0.01 ≥ P-value > 0.001; ***: 0.001 ≥ P-value. (DOCX 15 kb) [file 12863_2019_721_MOESM1_ESM.docx]

**Additional file 1. Pairwise genetic differentiation among *Impatiens glandulifera* populations.**

|  | **A1** | **A2** | **G1** | **G2** | **B1** | **B2** | **L1** | **L2** | **S1** | **S2** | **T1** | **T2** | **T3** |
| --- | --- | --- | --- | --- | --- | --- | --- | --- | --- | --- | --- | --- | --- |
| **A1** | 0.092^***^ | 0.057^***^ | 0.139^***^ | 0.195^***^ | 0.101^***^ | 0.094^***^ | 0.235^***^ | 0.189^***^ | 0.250^***^ | 0.295^***^ | 0.172^***^ | 0.339^***^ | 0.280^***^ |
| **A2** | 0.115^***^ | 0.026 ^NS^ | 0.176^***^ | 0.197^***^ | 0.107^***^ | 0.102^***^ | 0.278^***^ | 0.212^***^ | 0.307^***^ | 0.274^***^ | 0.230^***^ | 0.473^***^ | 0.303^***^ |
| **G1** | 0.093^***^ | 0.116^***^ | 0.007^NS^ | 0.082^***^ | 0.144^***^ | 0.115^***^ | 0.231^***^ | 0.210^***^ | 0.308^***^ | 0.280^***^ | 0.198^***^ | 0.319^***^ | 0.301^***^ |
| **G2** | 0.264^***^ | 0.212^***^ | 0.131^***^ | 0.032 ^NS^ | 0.198^***^ | 0.168^***^ | 0.288^***^ | 0.280^***^ | 0.343^***^ | 0.384^***^ | 0.286^***^ | 0.497^***^ | 0.411^***^ |
| **B1** | 0.097^***^ | 0.072^***^ | 0.138^***^ | 0.228^***^ | 0.027 ^*^ | 0.029 ^NS^ | 0.164^***^ | 0.184^***^ | 0.162^***^ | 0.137^***^ | 0.106^***^ | 0.201^***^ | 0.114^***^ |
| **B2** | 0.120^***^ | 0.077^***^ | 0.141^***^ | 0.296^***^ | 0.026 ^NS^ | 0.008 ^NS^ | 0.137^***^ | 0.159^***^ | 0.192^***^ | 0.130^***^ | 0.140^***^ | 0.238^***^ | 0.144^***^ |
| **L1** | 0.084^***^ | 0.194^***^ | 0.193^***^ | 0.374^***^ | 0.146^***^ | 0.189^***^ | 0.060^**^ | 0.035^*^ | 0.143^***^ | 0.125^***^ | 0.088^***^ | 0.130^***^ | 0.217^***^ |
| **L2** | 0.042^**^ | 0.056^***^ | 0.080^***^ | 0.173^***^ | 0.082^***^ | 0.087^***^ | 0.086^***^ | 0.088^***^ | 0.174^***^ | 0.159^***^ | 0.084^***^ | 0.131^***^ | 0.240^***^ |
| **S1** | 0.281^***^ | 0.373^***^ | 0.342^***^ | 0.462^***^ | 0.198^***^ | 0.242^***^ | 0.234^***^ | 0.287^***^ | 0.033^*^ | 0.118^***^ | 0.126^***^ | 0.112^***^ | 0.143^***^ |
| **S2** | 0.281^***^ | 0.307^***^ | 0.343^***^ | 0.503^***^ | 0.130^***^ | 0.177^***^ | 0.244^***^ | 0.233^***^ | 0.191^***^ | 0.029^*^ | 0.129^***^ | 0.197^***^ | 0.057^***^ |
| **T1** | 0.155^***^ | 0.220^***^ | 0.222^***^ | 0.357^***^ | 0.151^***^ | 0.174^***^ | 0.144^***^ | 0.135^***^ | 0.194^***^ | 0.174^***^ | 0.026^*^ | 0.032^*^ | 0.141^***^ |
| **T2** | 0.377^***^ | 0.567^***^ | 0.413^***^ | 0.694^***^ | 0.297^***^ | 0.394^***^ | 0.258^***^ | 0.317^***^ | 0.249^***^ | 0.303^***^ | 0.078^***^ | 0.055^***^ | 0.247^***^ |
| **T3** | 0.212^***^ | 0.270^***^ | 0.302^***^ | 0.397^***^ | 0.189^***^ | 0.217^***^ | 0.198^***^ | 0.195^***^ | 0.170^***^ | 0.137^***^ | 0.061^***^ | 0.078^***^ | 0.137^***^ |

Lower left triangle, F_ST_ estimates for 2011; Upper right triangle, F_ST_ estimates for 2016; values on the main diagonal (grey), F_ST_ estimates between 2011 and 2016 populations along a gradient from Amiens to Trondheim. A=Amiens, G=Ghent, B=Bremen, L=Lund, S=Stockholm, T=Trondheim. Significance: ^NS^: not significant; ^*^: 0.05 ≥ *P*-value > 0.01; ^**^: 0.01 ≥ *P*-value > 0.001; ^***^: 0.001 ≥ *P*-value.
