# Supplementary material for: No genetic erosion after five generations for Impatiens glandulifera populations across the invaded range in Europe
Source: BMC Genet. 2019 Feb 19;20:20. doi: 10.1186/s12863-019-0721-4 (PMC6379953; doi:10.1186/s12863-019-0721-4)
Supplement: Supplementary file 2 — Pairwise genetic differentiation among Impatiens glandulifera populations (G’ST). Lower left triangle, G’ST estimates for 2011; Upper right triangle, G’ST estimates for 2016; values on the main diagonal (grey), G’ST estimates between 2011 and 2016 populations along a gradient from Amiens to Trondheim. A = Amiens, G = Ghent, B=Bremen, L = Lund, S=Stockholm, T = Trondheim. Significance: NS: not significant; *: 0.05 ≥ P-value > 0.01; **: 0.01 ≥ P-value > 0.001; ***: 0.001 ≥ P-value. (DOCX 15 kb) [file 12863_2019_721_MOESM2_ESM.docx]

**Additional file 2. Pairwise genetic differentiation among *Impatiens glandulifera* populations.**

|  | **A1** | **A2** | **G1** | **G2** | **B1** | **B2** | **L1** | **L2** | **S1** | **S2** | **T1** | **T2** | **T3** |
| --- | --- | --- | --- | --- | --- | --- | --- | --- | --- | --- | --- | --- | --- |
| **A1** | 0.117^***^ | 0.116^***^ | 0.267^***^ | 0.398^***^ | 0.239^***^ | 0.263^***^ | 0.431^***^ | 0.434^***^ | 0.595^***^ | 0.527^***^ | 0.395^***^ | 0.573^***^ | 0.510^***^ |
| **A2** | 0.194^***^ | 0.046^**^ | 0.339^***^ | 0.410^***^ | 0.200^***^ | 0.213^***^ | 0.497^***^ | 0.502^***^ | 0.623^***^ | 0.460^***^ | 0.470^***^ | 0.645^***^ | 0.424^***^ |
| **G1** | 0.151^***^ | 0.249^***^ | -0.015 ^NS^ | 0.166^***^ | 0.292^***^ | 0.275^***^ | 0.400^***^ | 0.418^***^ | 0.631^***^ | 0.532^***^ | 0.398^***^ | 0.559^***^ | 0.548^***^ |
| **G2** | 0.413^***^ | 0.459^***^ | 0.278^***^ | 0.014 ^NS^ | 0.413^***^ | 0.392^***^ | 0.565^***^ | 0.573^***^ | 0.698^***^ | 0.615^***^ | 0.594^***^ | 0.714^***^ | 0.628^***^ |
| **B1** | 0.191^***^ | 0.114^***^ | 0.276^***^ | 0.463^***^ | 0.056^*^ | 0.015 ^NS^ | 0.340^***^ | 0.391^***^ | 0.409^***^ | 0.271^***^ | 0.285^***^ | 0.430^***^ | 0.230^***^ |
| **B2** | 0.220^***^ | 0.135^***^ | 0.297^***^ | 0.459^***^ | 0.013 ^NS^ | -0.007 ^NS^ | 0.323^***^ | 0.387^***^ | 0.459^***^ | 0.252^***^ | 0.344^***^ | 0.496^***^ | 0.270^***^ |
| **L1** | 0.124^***^ | 0.411^***^ | 0.376^***^ | 0.616^***^ | 0.304^***^ | 0.366^***^ | 0.068^**^ | 0.035^*^ | 0.374^***^ | 0.276^***^ | 0.145^***^ | 0.264^***^ | 0.417^***^ |
| **L2** | 0.062^***^ | 0.104^***^ | 0.159^***^ | 0.409^***^ | 0.187^***^ | 0.216^***^ | 0.208^***^ | 0.239^***^ | 0.439^***^ | 0.372^***^ | 0.159^***^ | 0.293^***^ | 0.489^***^ |
| **S1** | 0.524^***^ | 0.615^***^ | 0.670^***^ | 0.745^***^ | 0.451^***^ | 0.488^***^ | 0.509^***^ | 0.561^***^ | 0.041^*^ | 0.321^***^ | 0.285^***^ | 0.287^***^ | 0.320^***^ |
| **S2** | 0.465^***^ | 0.444^***^ | 0.608^***^ | 0.709^***^ | 0.239^***^ | 0.289^***^ | 0.475^***^ | 0.479^***^ | 0.357^***^ | 0.028^*^ | 0.293^***^ | 0.371^***^ | 0.074^***^ |
| **T1** | 0.301^***^ | 0.433^***^ | 0.429^***^ | 0.672^***^ | 0.348^***^ | 0.415^***^ | 0.299^***^ | 0.293^***^ | 0.398^***^ | 0.363^***^ | 0.037^*^ | 0.037^*^ | 0.300^***^ |
| **T2** | 0.576^***^ | 0.729^***^ | 0.688^***^ | 0.858^***^ | 0.573^***^ | 0.642^***^ | 0.548^***^ | 0.609^***^ | 0.467^***^ | 0.479^***^ | 0.125^***^ | 0.087^***^ | 0.366^***^ |
| **T3** | 0.462^***^ | 0.550^***^ | 0.598^***^ | 0.751^***^ | 0.441^***^ | 0.502^***^ | 0.445^***^ | 0.452^***^ | 0.377^***^ | 0.279^***^ | 0.080^***^ | 0.115^***^ | 0.271^***^ |

Lower left triangle, G’_ST_ estimates for 2011; Upper right triangle, G’_ST_ estimates for 2016; values on the main diagonal (grey), G’_ST_ estimates between 2011 and 2016 populations along a gradient from Amiens to Trondheim. A=Amiens, G=Ghent, B=Bremen, L=Lund, S=Stockholm, T=Trondheim. Significance: ^NS^: not significant; ^*^: 0.05 ≥ *P*-value > 0.01; ^**^: 0.01 ≥ *P*-value > 0.001; ^***^: 0.001 ≥ *P*-value.
