# Supplementary material for: No genetic erosion after five generations for Impatiens glandulifera populations across the invaded range in Europe
Source: BMC Genet. 2019 Feb 19;20:20. doi: 10.1186/s12863-019-0721-4 (PMC6379953; doi:10.1186/s12863-019-0721-4)
Supplement: Supplementary file 3 — Pairwise genetic differentiation among Impatiens glandulifera populations (Jost’s D). Lower left triangle, Jost’s D estimates for 2011; Upper right triangle, Jost’s D estimates for 2016; values on the main diagonal (grey), Jost’s D estimates between 2011 and 2016 populations along a gradient from Amiens to Trondheim. A = Amiens, G = Ghent, B=Bremen, L = Lund, S=Stockholm, T = Trondheim. Significance: NS: not significant; *: 0.05 ≥ P-value > 0.01; **: 0.01 ≥ P-value > 0.001; ***: 0.001 ≥ P-value. (DOCX 15 kb) [file 12863_2019_721_MOESM3_ESM.docx]

**Additional file 3. Pairwise genetic differentiation among *Impatiens glandulifera* populations.**

|  | **A1** | **A2** | **G1** | **G2** | **B1** | **B2** | **L1** | **L2** | **S1** | **S2** | **T1** | **T2** | **T3** |
| --- | --- | --- | --- | --- | --- | --- | --- | --- | --- | --- | --- | --- | --- |
| **A1** | 0.043^***^ | 0.033^***^ | 0.114^***^ | 0.175^***^ | 0.107^***^ | 0.113^***^ | 0.197^***^ | 0.176^***^ | 0.320^***^ | 0.261^***^ | 0.187^***^ | 0.284^***^ | 0.253^***^ |
| **A2** | 0.075^***^ | 0.012^**^ | 0.147^***^ | 0.173^***^ | 0.084^***^ | 0.084^***^ | 0.231^***^ | 0.207^***^ | 0.332^***^ | 0.203^***^ | 0.228^***^ | 0.334^***^ | 0.184^***^ |
| **G1** | 0.067^***^ | 0.103^***^ | -0.006^NS^ | 0.072^***^ | 0.155^***^ | 0.138^***^ | 0.207^***^ | 0.201^***^ | 0.399^***^ | 0.306^***^ | 0.217^***^ | 0.317^***^ | 0.324^***^ |
| **G2** | 0.188^***^ | 0.182^***^ | 0.115^***^ | 0.004^NS^ | 0.227^***^ | 0.202^***^ | 0.318^***^ | 0.298^***^ | 0.452^***^ | 0.361^***^ | 0.362^***^ | 0.454^***^ | 0.380^***^ |
| **B1** | 0.093^***^ | 0.047^***^ | 0.145^***^ | 0.242^***^ | 0.018^*^ | 0.007^NS^ | 0.177^***^ | 0.196^***^ | 0.224^***^ | 0.134^***^ | 0.151^***^ | 0.229^***^ | 0.112^***^ |
| **B2** | 0.101^***^ | 0.050^***^ | 0.146^***^ | 0.218^***^ | 0.006 ^NS^ | -0.003^NS^ | 0.159^***^ | 0.182^***^ | 0.249^***^ | 0.116^***^ | 0.181^***^ | 0.267^***^ | 0.128^***^ |
| **L1** | 0.046^***^ | 0.164^***^ | 0.176^***^ | 0.302^***^ | 0.144^***^ | 0.166^***^ | 0.023^**^ | 0.012^*^ | 0.177^***^ | 0.120^***^ | 0.062^***^ | 0.107^***^ | 0.207^***^ |
| **L2** | 0.025^**^ | 0.036^***^ | 0.068^***^ | 0.175^***^ | 0.087^***^ | 0.094^***^ | 0.078^***^ | 0.091^***^ | 0.198^***^ | 0.156^***^ | 0.062^***^ | 0.108^***^ | 0.236^***^ |
| **S1** | 0.309^***^ | 0.356^***^ | 0.457^***^ | 0.496^***^ | 0.267^***^ | 0.279^***^ | 0.268^***^ | 0.330^***^ | 0.016^*^ | 0.143^***^ | 0.133^***^ | 0.118^***^ | 0.145^***^ |
| **S2** | 0.240^***^ | 0.194^***^ | 0.366^***^ | 0.414^***^ | 0.113^***^ | 0.129^***^ | 0.217^***^ | 0.239^***^ | 0.171^***^ | 0.010^*^ | 0.137^***^ | 0.163^***^ | 0.028^***^ |
| **T1** | 0.145^***^ | 0.202^***^ | 0.231^***^ | 0.398^***^ | 0.187^***^ | 0.217^***^ | 0.126^***^ | 0.134^***^ | 0.209^***^ | 0.170^***^ | 0.015^*^ | 0.014^*^ | 0.144^***^ |
| **T2** | 0.275^***^ | 0.363^***^ | 0.391^***^ | 0.548^***^ | 0.303^***^ | 0.335^***^ | 0.209^***^ | 0.287^***^ | 0.202^***^ | 0.181^***^ | 0.038^***^ | 0.022^***^ | 0.163^***^ |
| **T3** | 0.240^***^ | 0.272^***^ | 0.359^***^ | 0.471^***^ | 0.243^***^ | 0.270^***^ | 0.200^***^ | 0.222^***^ | 0.185^***^ | 0.114^***^ | 0.030^**^ | 0.031^***^ | 0.116^***^ |

Lower left triangle, Jost’s D estimates for 2011; Upper right triangle, Jost’s D estimates for 2016; values on the main diagonal (grey), Jost’s D estimates between 2011 and 2016 populations along a gradient from Amiens to Trondheim. A=Amiens, G=Ghent, B=Bremen, L=Lund, S=Stockholm, T=Trondheim. Significance: ^NS^: not significant; ^*^: 0.05 ≥ *P*-value > 0.01; ^**^: 0.01 ≥ *P*-value > 0.001; ^***^: 0.001 ≥ *P*-value.
