# Supplementary material for: No genetic erosion after five generations for Impatiens glandulifera populations across the invaded range in Europe
Source: BMC Genet. 2019 Feb 19;20:20. doi: 10.1186/s12863-019-0721-4 (PMC6379953; doi:10.1186/s12863-019-0721-4)
Supplement: Supplementary file 4 — Pairwise genetic differentiation among Impatiens glandulifera populations (Null-allele corrected FST). Lower left triangle, null-allele corrected FST estimates for 2011; Upper right triangle, null-allele corrected FST estimates for 2016; values on the main diagonal (grey), null-allele corrected FST estimates between 2011 and 2016 populations along a gradient from Amiens to Trondheim. A = Amiens, G = Ghent, B=Bremen, L = Lund, S=Stockholm, T = Trondheim. (DOCX 14 kb) [file 12863_2019_721_MOESM4_ESM.docx]

**Additional file 4. Pairwise genetic differentiation among *Impatiens glandulifera* populations.**

|  | **A1** | **A2** | **G1** | **G2** | **B1** | **B2** | **L1** | **L2** | **S1** | **S2** | **T1** | **T2** | **T3** |
| --- | --- | --- | --- | --- | --- | --- | --- | --- | --- | --- | --- | --- | --- |
| **A1** | 0.228 | 0.274 | 0.295 | 0.354 | 0.302 | 0.362 | 0.456 | 0.407 | 0.534 | 0.555 | 0.390 | 0.526 | 0.458 |
| **A2** | 0.461 | 0.195 | 0.334 | 0.405 | 0.204 | 0.276 | 0.469 | 0.449 | 0.602 | 0.594 | 0.507 | 0.577 | 0.407 |
| **G1** | 0.233 | 0.283 | 0.019 | 0.213 | 0.263 | 0.309 | 0.369 | 0.358 | 0.527 | 0.539 | 0.394 | 0.491 | 0.455 |
| **G2** | 0.237 | 0.332 | 0.072 | 0.048 | 0.358 | 0.373 | 0.488 | 0.458 | 0.581 | 0.594 | 0.512 | 0.604 | 0.498 |
| **B1** | 0.188 | 0.378 | 0.252 | 0.236 | 0.062 | 0.069 | 0.300 | 0.323 | 0.449 | 0.458 | 0.345 | 0.364 | 0.219 |
| **B2** | 0.447 | 0.590 | 0.402 | 0.457 | 0.348 | 0.020 | 0.315 | 0.352 | 0.532 | 0.512 | 0.465 | 0.473 | 0.290 |
| **L1** | 0.150 | 0.523 | 0.274 | 0.344 | 0.349 | 0.549 | 0.110 | 0.063 | 0.480 | 0.506 | 0.347 | 0.279 | 0.389 |
| **L2** | 0.108 | 0.440 | 0.205 | 0.274 | 0.241 | 0.390 | 0.204 | 0.217 | 0.476 | 0.517 | 0.307 | 0.289 | 0.408 |
| **S1** | 0.551 | 0.632 | 0.411 | 0.507 | 0.577 | 0.638 | 0.513 | 0.525 | 0.035 | 0.319 | 0.281 | 0.309 | 0.397 |
| **S2** | 0.538 | 0.588 | 0.300 | 0.438 | 0.572 | 0.633 | 0.503 | 0.490 | 0.328 | 0.054 | 0.358 | 0.387 | 0.352 |
| **T1** | 0.451 | 0.530 | 0.311 | 0.468 | 0.469 | 0.603 | 0.380 | 0.381 | 0.411 | 0.383 | 0.048 | 0.098 | 0.339 |
| **T2** | 0.619 | 0.701 | 0.493 | 0.581 | 0.611 | 0.791 | 0.560 | 0.622 | 0.489 | 0.458 | 0.173 | 0.173 | 0.363 |
| **T3** | 0.451 | 0.622 | 0.363 | 0.470 | 0.529 | 0.641 | 0.428 | 0.391 | 0.458 | 0.401 | 0.274 | 0.337 | 0.284 |

Lower left triangle, null-allele corrected F_ST_ estimates for 2011; Upper right triangle, null-allele corrected F_ST_ estimates for 2016; values on the main diagonal (grey), null-allele corrected F_ST_ estimates between 2011 and 2016 populations along a gradient from Amiens to Trondheim. A=Amiens, G=Ghent, B=Bremen, L=Lund, S=Stockholm, T=Trondheim.
